# Challenges in the Management of Post-COVID-19 Pulmonary Fibrosis for the Latin American Population

**DOI:** 10.3390/jpm12091393

**Published:** 2022-08-27

**Authors:** Ivan Cherrez-Ojeda, Arturo Cortés-Telles, Laura Gochicoa-Rangel, Génesis Camacho-Leon, Hans Mautong, Karla Robles-Velasco, Marco Faytong-Haro

**Affiliations:** 1 School of Health, Universidad de Especialidades Espíritu Santo, Samborondón 0901952, Guayas, Ecuador; 2 Respiralab Research Group, Guayaquil 090512, Guayas, Ecuador; 3 Departamento de Neumología y Cirugía de Tórax, Hospital Regional de Alta Especialidad de Yucatán, Mérida 97133, Mexico; 4 Department of Respiratory Physiology, National Institute of Respiratory Diseases “Ismael Cosío Villegas”, Mexico City 14080, Mexico; 5 Division of Clinical and Translational Research, Larkin Community Hospital, South Miami, FL 33143, USA; 6 Sociology and Demography Department, The Pennsylvania State University, University Park, PA 16802, USA; 7 Ecuadorian Development Research Lab, Daule 090656, Guayas, Ecuador

**Keywords:** COVID-19, Latin America, pulmonary fibrosis, pandemic, SARS-CoV-2

## Abstract

This commentary aims to highlight some of the major issues (with possible solutions) that the Latin American region is currently dealing with in managing post-COVID-19 pulmonary fibrosis. Overall, there is little evidence for successful long-term COVID-19 follow-up treatment. The lack of knowledge regarding proper treatment is exacerbated in Latin America by a general lack of resources devoted to healthcare, and a lack of availability and access to multidisciplinary teams. The discussion suggests that better infrastructure (primarily multicenter cohorts of COVID-19 survivors) and well-designed studies are required to develop scientific knowledge to improve treatment for the increasing prevalence of pulmonary fibrosis in Latin America.

## 1. Introduction

New highly pathogenic viruses such as coronaviruses (CoVs) crossing the animal–human barrier remain a global health threat [1]. The last emerging zoonosis was identified in 2019 with the first case of a severe acute respiratory syndrome caused by a newly detected coronavirus, the SARS-CoV-2. Since then, it has spread worldwide [2].

The COVID-19 pandemic has affected 268 million people worldwide, including more than 50 million in Latin America. This region has been disproportionately affected by the COVID-19 pandemic, and many countries still struggle to contain the virus. Comprising 8% of the global population, the region has borne 20% of infections and 32% of cumulative global deaths [3]. A meta-analysis published in 2021 by Thakur et al. [4] of 125,546 patients showed that patients from Latin American countries had the highest mortality compared with the rates of the European countries hardest hit by COVID-19. Brazil had the highest number of confirmed cases in the region, with 22.29 million confirmed cases since the beginning of the pandemic, followed by Argentina with 5.7 million. Countries such as Colombia, Peru, Chile, and Ecuador have also had high numbers of COVID-19 cases [5].

Moreover, multiple respiratory symptoms were reported to persist after acute infection, including dyspnea, fatigue, and effort intolerance, in up to 40% of cases in a 1-year follow-up [6]. As the number of recovered patients increases, acquiring information about pulmonary sequelae, which can persist or develop after the initial recovery, is necessary. However, to our knowledge, there are no prevalence studies of post-COVID-19 pulmonary fibrosis (PCPF) in Latin America. Diagnosis involves persistent respiratory symptoms accompanied by abnormalities in pulmonary function tests and imaging results from serial chest computed tomography [7,8]. No fully proven options are available for the treatment of PCPF. However, to identify the most appropriate potential therapeutic target, clinical trials involving antifibrotics, corticosteroids, and new molecules are under development [9,10,11,12,13].

Latin America is a region that has been dealing with slow economic growth for years, even before the pandemic, and many of its countries face poverty [14], inequality of many types [15,16,17], and vulnerability [18]. According to studies by Liang et al., mortality from COVID-19 was negatively associated with COVID-19 test number per 100 people, government effectiveness score, and hospital bed number [19]. Their results also showed a significant difference in the average testing rate, which indicates that the Latin American population tested almost three times less than countries in other regions of the world [20].

An impact of the pandemic in Latin America is post-COVID-19 syndrome (PCS), a sequela that causes persistent symptoms more than 3 months after infection. Because there are no effective methods for assessing PCS, its causes and public health impact are unclear [21]. Considering the alarming number of individuals infected with SARS-CoV-2, we should anticipate an increase in the incidence of long-term pulmonary sequelae and a non-negligible proportion of post-COVID-19 pulmonary disease, including possible fibrotic changes [22].

## 2. Post-COVID-19 Pulmonary Fibrosis

For survivors of moderate to severe COVID-19 illness, defeating the virus is only the start of a new path to recovery. What occurs following the acute phase of SARS-CoV-2 infection is contingent on the extent and severity of viral attacks on various cell and organ types. The term “long COVID” has been used to describe a range of new or persistent signs and symptoms that can last weeks to months after acute COVID-19, and can worsen with physical or mental activity [22]. This health problem may be due to the persistence of residual inflammation or immune response, repair, and remodeling mechanisms, which could influence the development of new lung lesions or the worsening of pre-existing ones [23]. It should be noted that other coronaviruses have been previously described as one of the many causative agents that can lead to the development of interstitial lung disease [24].

Similar patterns of fibrosis with deficits in pulmonary function have been described in patients infected with these viruses [25], which may set the tone for identifying SARS-CoV-2-related pulmonary conditions. Long COVID is a type of post-COVID-19 syndrome that can include patients with evidence of fibrotic-like changes following an acute COVID-19 infection. As a result, this may lead to a significant future burden in Latin America, where many people have been infected [26].

The natural history of long COVID and its pulmonary fibrotic sequelae are not well understood yet. Clinical and radiological abnormalities linked to this post-COVID-19 condition have been shown to last for at least 6 months in at least one-third of patients that suffered SARS-CoV-2 infection [27]. These events are most pronounced for elderly patients who may have had subclinical interstitial lung disease [28]. These elderly patients are also more likely to die from COVID-19 complications [29].

Furthermore, a subset of patients could present with post-COVID-19 pulmonary fibrosis, which may be defined as the presence of persistent fibrotic tomographic sequelae associated with functional impairment [27].

### 2.1. Pathophysiology

Merza et al. reported that inflammatory immune cells, proinflammatory cytokines, and adhesion molecules are crucial factors in acute lung injury. It has been described as a severe-SARS-CoV-2-pneumonia cytokine storm process that creates a state of hyperinflammation involving proinflammatory cytokines such as TNF-α, IL-1, IL-8, and IL-6, among others [30]. This histologically severe pulmonary injury has been described as diffused alveolar damage (DAD), which is the hallmark of severe COVID-19, and can have two phases: the acute/exudative phase and the second organizational/proliferative phase [28].

The first phase is characterized by prominent reactive pneumocytes, pulmonary hemorrhage, intra-alveolar fibrin deposition, interstitial edema, hyaline membrane, giant cell formation, and bronchiolitis. Furthermore, thrombotic events in the pulmonary arteries can occur in this phase due to inflammatory influences such as inflammasomes and extracellular neutrophil traps [29]. During the second phase, the basement membrane ruptures, thickening the alveolar wall as a result of organizing fibroblast proliferation and prominent, squamous metaplasia of type II pneumocytes [31]. After this second stage, there may be progression to the fibrosing phase, with excessive extracellular matrix and collagen deposition and diffused thickening of the alveolar walls. This can determine the distortion of the lung architecture, similar to patterns of non-specific cellular and fibrosing interstitial pneumonitis [28].

In a series of 30 patients who underwent a minimally invasive autopsy, the most common finding was DAD (93%), with acute (40%), organizational (70%), or fibrosing patterns (isolated or combined). Fibrosing DAD implicates the development of long-term post-COVID-19 pulmonary fibrosis [28]. COVID-19 patients have also been reported to develop virus-induced organizing pneumonia. This histological pattern implies that the alveoli and alveolar ducts fill with fibroblasts and myofibroblasts as an inflammatory response to the presence of the virus. This pattern resolves naturally or can become chronic due to excessive collagen deposition and progress to pulmonary fibrosis—fortunately, treatment with corticosteroids seems to alleviate this case of pulmonary fibrosis [32].

Although there is no official consensus, post-COVID-19 pulmonary fibrosis comprises fibrotic-like sequelae, such as organizing pneumonia, interstitial lung disease, or pulmonary fibrosis, after a COVID-19 infection. A recent study among survivors of severe COVID-19 with lung sequelae revealed that 56% had lung “fibrotic-like” changes at 1-year follow-up according to their computerized tomography scans (CTs) [27]. According to this, post-COVID-19 pulmonary fibrosis prevalence could be 10 to 15 patients per 10,000 people, much higher than the prevalence of idiopathic pulmonary fibrosis (IPF) [33].

Unfortunately, current data do not offer reliable rates of COVID-19-induced fibrosis. Nevertheless, Bazdyrev et al. [33] estimated that the risk of developing pulmonary fibrosis after COVID-19 might be 2–6% after moderate/severe illness. It is unclear whether fibrotic-like CT features represent irreversible disease or a slowly regressive infiltrate secondary to organizing pneumonia, as seen with mild distortion mimicking actual fibrosis. The clinical management of long COVID and its subsets, such as post-COVID-19 pulmonary fibrosis, is challenging due to a lack of evidence-based guidelines and standardization in the pulmonary definition/terminology of the post-COVID-19 condition.

**Figure 1:**
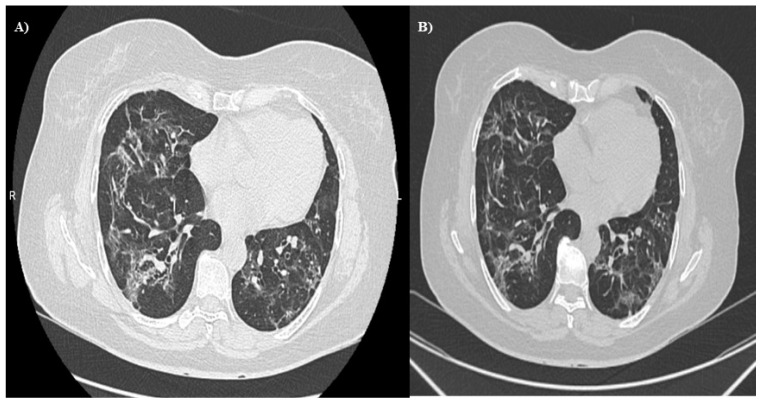
CT scan showing abnormalities in the lungs of one patient following severe COVID-19 infection. (**A**) Patient is 71 years old, 1-year follow-up post-COVID-19. Bilateral cobblestone lung images predominantly in the upper lobes and the upper segments of the lower lung lobes are associated with subpleural dense linear images. (**B**) Same patient as Figure 1A. Two-year follow-up post-COVID-19. Pulmonary images of cobblestone pattern in the upper lobes and the upper segments of the lower lobes are associated with images of traction bronchiectasis. Septal thickening in the lower lobes is associated with subpleural dense linear images.

Cherrez et al. [7] have reported the follow-up of two cases of pulmonary fibrosis after severe COVID-19 infection. One year later, both cases maintained symptoms of shortness of breath upon exertion and persistence of ground-glass-type opacities, traction bronchiectasis, and interlobular septal thickening that covered much of the lung parenchyma (Figure 1A,B)

### 2.2. Risk Factors

**Figure 2:**
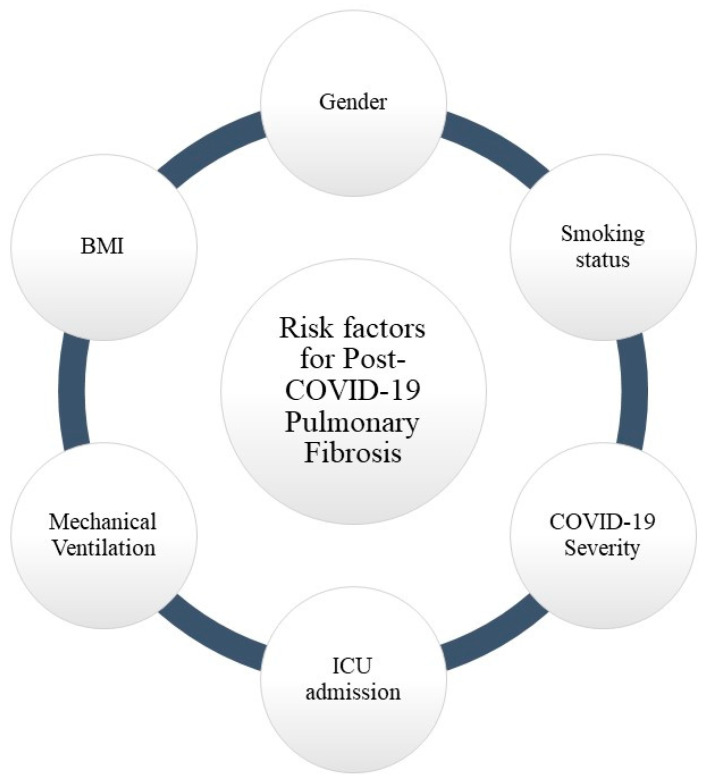
Possible risk factors for post-COVID-19 pulmonary fibrosis.

Although the pathophysiology and progression of post-COVID-19 pulmonary fibrosis are unknown, it is thought to have multiple risk factors (that trigger a storm of cytokines that damage the pulmonary endothelium and parenchyma, causing dysfunction [8]. Figure 2 shows the possible risk factors of PCPF.

First, gender is a potential PCPF risk factor. Many studies have evidenced that male patients tend to have an increased prevalence of post-COVID-19 pulmonary fibrosis. However, many other cohort studies have not shown a significant increase in the risk of developing fibrosis [8]. The severity of COVID-19, on the other hand, has been extensively reported as a significant risk factor for post-COVID-19 pulmonary fibrosis. Interestingly, being male is strongly associated with developing severe acute COVID-19, thus making gender a possible confounder that distorts the relationship between COVID-19 severity and the development of fibrosis [34]. Smoking status has also been identified as a strong risk factor for post-COVID-19 pulmonary fibrosis [34,35].

Mechanical ventilation and a prolonged stay in the ICU (Intensive Care Unit) appear to be factors that eventually cause changes in lung parenchymal structure. It has been reported that 5–12% of COVID-19 cases require ICU care; however, it has also been proposed that the use of mechanical ventilation is an additional risk factor for the development of pulmonary fibrosis, and that variations in the volumes used in mechanically ventilated patients, have increased the risk of developing acute respiratory distress syndrome (ARDS) due to the spread of proinflammatory factors, and thus, increased mortality [36]. Furthermore, a BMI (Body Mass Index) greater than 29 may be considered a risk factor, as it frequently correlates with the presence of metabolic syndrome, hypertension, coronary heart disease, and diabetes, all of which alter the immune response, potentially activating the proinflammatory cascade and correlating with an increase in disease severity [36]. Finally, it is difficult to determine whether the fibrotic-like changes observed result from a single risk factor or a combination of risk factors, and whether the changes are irreversible. Periodic follow-ups are required to answer these questions [37].

Since post-COVID-19 pulmonary fibrosis is a new clinical entity, significant research efforts are being conducted to establish its pathophysiology, risk factors, diagnosis, and management. Most of the information available on this disease comes from developed countries. This commentary highlights some of the critical challenges Latin America is currently facing in managing post-COVID-19 pulmonary fibrosis, since this population has been profoundly affected by the COVID-19 pandemic [38]. Overall, the evidence for long-term COVID-19 follow-up care is limited; however, the following sections describe the challenges, existing treatments, and options currently under study.

## 3. Challenges in Monitoring and Rehabilitation

Persistent inflammatory and immune reactions in the post-COVID-19 period may play a role in the development of lung lesions. Although some patients recover from severe COVID-19 pneumonia, they remain symptomatic in the post-infectious period, with clinical, radiological, or respiratory abnormalities despite a negative SARS-CoV-2 control test [39]. The persistence of residual inflammation or immune response, repair, and remodeling mechanisms in the post-COVID-19 period may influence the development of new lung lesions, or the worsening of pre-existing lesions. There are many hypotheses around the development of long-term pulmonary fibrosis in this group of patients, and with it, a potentially enormous burden of future morbidity in the population [26].

The follow-up of long COVID-19 is based on the European Respiratory Society (ERS) statement. It is expected that 25% of survivors of ARDS will manifest restrictive lung disease on PFTs (pulmonary function tests) 6 months after the diagnosis [40]. As a result, a follow-up evaluation 1 to 6 months after the acute infection is recommended, particularly for severe COVID-19 cases [41]. Pulmonary function tests (PFTs) are essential in evaluating the respiratory system. In this regard, it is imperative to have a PFT system that enables a complete evaluation, reviews the efforts made by the interpreting physician, and reports results [42]. The risk factors that are used to predict an abnormal diffusing capacity of the lungs for carbon monoxide (DLCO) and pulmonary function (and, therefore, determine the urgency for follow-up) are age, COVID-19 severity (oxygenation and ventilation modality, and radiological data), biological parameters (D-dimer, T cell count, LDH, IL-6), and ARDS.

Computerized tomography (CT) seems to be the most accurate method for severity assessment [43], as well as the monitoring of “fibrotic-like” features. However, it is unclear up to this point whether the latter is irreversible or slowly regressive. Reduced DLCO seems to be the most sensitive impairment in pulmonary function tests (PFT) [44]. Total lung capacity and expiratory rates are valuable tools for monitoring long COVID-19.

The most common pulmonary symptom after acute COVID-19 infection is dyspnea, according to multiple reports in the literature, which can affect up to half of the infected patients, regardless of the severity of the initial infection [45,46]. In addition, pulmonary physiology is affected, and various pulmonary function tests can detect this. These tests are classified depending on the parameter they assess. Respiratory mechanics are measured by spirometry, impulse oscillometry, forced oscillometry, plethysmography, multiple breathing washout techniques (MBW), and maximum inspiratory and expiratory pressure (MiP and MeP, respectively).

For gas exchange assessment, a carbon monoxide diffusion test, oximetry, blood gas analysis, and a six-minute walk test are performed [47]. In addition, ventilatory control tests and inflammatory biomarkers (including exhaled fraction of nitric oxide, exhaled nitric oxide, or volatile organic compounds), not to mention imaging tests (such as high-resolution tomography with inspiration and expiration slices), are also used. Anomalies in any of these diagnostic techniques can range from restrictive pulmonary processes, DLCO imbalance, persistent desaturation, and tomographic abnormalities that remain up to 2 years post-COVID-19, according to the literature [48,49]. These findings in a post-COVID-19 patient correlate with the possibility that the patient requires oxygen or additional treatments to cope with their pulmonary recovery or stabilization process [33].

The ERS statement on long COVID-19 emphasizes the importance of DLCO and TLC (total lung capacity) in assessing long-term functional lung sequelae. However, Latin America faces severe challenges as DLCO, the central core of COVID-19 follow-up, is unavailable in several respiratory medicine centers [50]. TLC is measured through a plethysmograph, which is even more complex and not widely distributed in the region. For these reasons, more cost-effective measures, such as the six-minute walk test (6MWT), may become more critical in developing countries.

Beyond the moderate and severe pneumonia-related fibrosis caused by the SARS-CoV-2 virus, there is a new pattern called post-COVID-19 small airway disease that is not well described in the literature. This finding could be related to the long-term evolution of the patients [51]. Suspicion of this pattern should be kept in mind during air trapping in serial inspiratory/expiratory thin-section CT (computerized tomography) [52]. Expectedly, the diagnosis could go unnoticed when performing spirometric tests. However, there are other methods of assessing the most distal airway, such as the MBW (multiple breathing washout) with its indicators, namely LCI (lung clearance index) and FRC (functional residual capacity), and secondly, oscillometry using the forced oscillation technique (FOT) [53,54].

**Figure 3:**
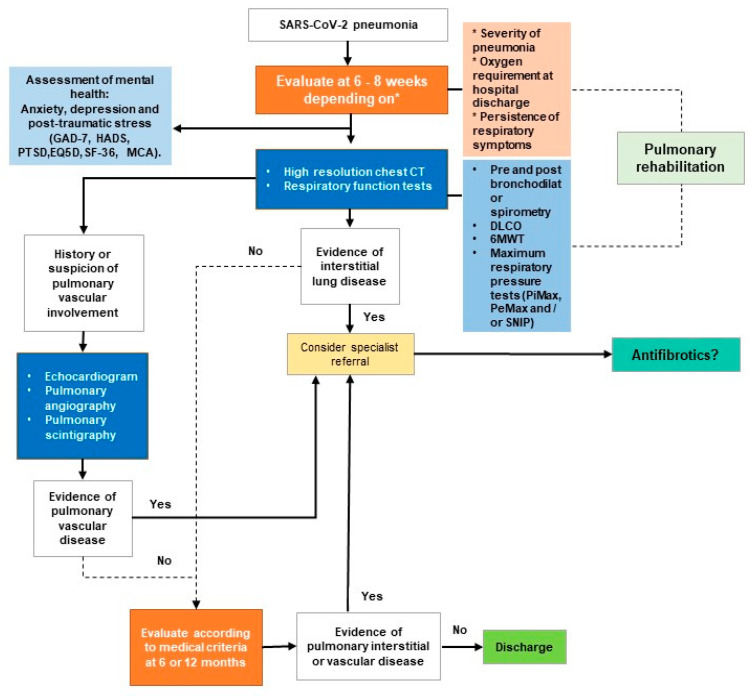
Model of a comprehensive post-COVID-19 monitoring program, elaborated by authors. Colors in the graph differ only for aesthetic reasons. Asterisk (*) is there to signal what criteria SARS-CoV-2 depend on.

The FOT is the least common of these diagnostic tools in Latin American countries. Ecuador was one of the first countries in South America to possess the equipment to conduct the FOT—there are only two instruments in the whole country. As a result of the limited supply of diagnostic options and the high demand from patients, post-COVID-19 functional respiratory patterns emerging as research progresses around the world may go undiagnosed. Based on the follow-up strategies in this section, Figure 3 shows a model elaborated by the authors for a comprehensive post-COVID-19 monitoring program.

## 4. Ongoing Studies on Medication as Treatment

According to Amin et al. [8], about 44% of COVID survivors develop post-COVID-19 pulmonary fibrosis. Therefore, a pulmonary rehabilitation plan and the development of new therapeutic strategies is needed. However, current strategies to reduce the severity and progression of post-COVID-19 pulmonary fibrosis are unclear. There is no standardized therapeutic approach to managing pulmonary fibrosis after SARS-CoV-2. Nevertheless, therapies such as corticosteroids, spironolactone, and fibrinolytic and antiviral agents have been proposed [55,56]. Patients with autoinflammatory phenotypes may benefit from steroid therapy. Early treatment was well tolerated and associated with rapid and significant subjective improvement in breathlessness and objective improvements in lung function in patients with persistent interstitial lung disease after COVID-19 [57,58]. However, a subset of patients do not respond well to steroids, which raises the question of whether other therapies, such as antifibrotics, could play a role in this circumstance. Antifibrotics mainly target the TGF-β pathway. Table 1 summarizes potential therapeutic antifibrotics in post-COVID-19 pulmonary fibrosis.

As the pathophysiological mechanisms of pulmonary fibrosis are similar to those of COVID-19 infection, it has been suggested that IPF regimens may be beneficial for treating COVID-19 pneumonia. A clinical rationale for using antifibrotic therapy in COVID-19 patients is to prevent complications of ongoing infection, stimulate the recovery phase, and control fibroproliferative processes [64].

The most studied antifibrotics are Nintedanib and Pirfenidone, which are widely used in idiopathic pulmonary fibrosis (IPF), and have shown reductions of up to 50% in the risk of loss of lung function, and increases in life expectancy of 2.5 years [65,66]. Their mechanism of action is based on the inhibition of mediators and profibrotic signaling to reduce the proliferation, migration, and differentiation of fibroblasts in the pulmonary extracellular matrix. Pirfenidone has a similar mechanism with an additional function, its ability to regulate proinflammatory cytokines, which adds to its effect on lung cancer.

Several ongoing studies on ClinicalTrials.gov aim to assess the use of antifibrotics to prevent/attenuate the progression of pulmonary fibrosis after COVID-19. The results of these trials could potentially lead to an individualized and tailored antifibrotic therapy. Regarding Pirfenidone, there are three clinical trials, all conducted in the patient recruitment phase, to evaluate the effect of this drug in patients with pulmonary fibrotic changes after having suffered severe pneumonia due to COVID-19. The clinical trials are: FIBRO-COVID NCT04607928 [9], PINCER NCT04856111 [10], and NCT04652518 [11]. The last one used Deupirfenidone, the chemical variant of Pirfenidone, to reduce the gastrointestinal discomfort of the original molecule.

Three clinical trials evaluate Nintedanib’s effectiveness in treating pulmonary fibrosis patients with moderate and severe disease due to COVID-19. They are NCT04338802 [12], ENDCOV-I NCT04619680 [13], and the previously mentioned PINCER study [10]. In addition to these antifibrotics, 12 more molecules are under study to evaluate their effectiveness as potential therapeutic agents in post-COVID-19 pulmonary fibrosis. One of them is the use of an intravenous form of mineralocorticoid receptor antagonist canrenoate potassium (an aldosterone antagonist of the spironolactone group) (NCT04912011) [67]; spironolactone or bovhyaluronidase azoxymer 3000 IU intramuscularly once every 5 days with a course of 15 injections (NCT04645368) [68]; MON002, which is an autologous monocyte product, cultured in vitro prior to intravenous delivery into patients with post-COVID-19 lung fibrosis (NCT04805086) [69]; BIO300, which is a suspension of genistein nanoparticles (NCT04482595) [70]; the autologous cellular stromal vascular fraction (cSVF) deployed intravenously (NCT04326036) [13]; Treamid drug taken at a dose of 50 mg (NCT04527354) [68]. Colchicine (NCT04818489) is an anti-inflammatory drug that inhibits the synthesis of TNF-α, IL-6, monocyte migration, and secretion of matrix metalloproteinase-9. These effects diminish the fibrotic impact of COVID-19 in severe cases [61].

The use of Prednisone 20 mg for 14 days has also been proposed as an option to manage post-COVID-19 pulmonary fibrosis (NCT04551781) [71]. Regarding traditional Chinese medicine, Fuzheng Huayu tablets (FZHY) at a dose of 1.6 g three times/day are in clinical trials to identify their efficacy and safety in post-COVID-19 patients with pulmonary fibrosis (NCT04279197) [63]. Mesenchymal stem cell (MSC)-based infusion therapies named EV-Pure™ and WJ-Pure™ are alternative treatments for COVID-19-induced pulmonary fibrosis (NCT05387239) [72]. Finally, the IN01 vaccine, which is a biological component that contains in its structure a protein possessing the EGF (epidermal growth factor) portion EGF-4-EGF with a subdomain of the B subunit of the cholera toxin. Therefore, once inoculated, it induces anti-EGF polyclonal neutralizing antibodies, thereby preventing the binding of this factor to its receptors, blocking the signaling of pathways related to the growth of tumor growth factor and fibrosis (NCT04537130) [73]. The scientific community is still awaiting the results of these studies.

Serum biomarkers of pulmonary fibrosis are classified according to the mechanism driving fibroproliferation: alveolocyte damage, including the Krebs von den Lungen antigen (KL-6), surfactant proteins A and D (SP-A, SP-D), and chitinase-like protein (YKL-40); fibrogenesis and fibroproliferation; matrix remodeling, including matrix metalloproteinases 1 and 7 (MMP1, MMP7); and vascular mechanisms (IL-8). Despite many past or ongoing studies, specific criteria for evaluating post-COVID-19 pulmonary fibrosis have not been established [40].

A study showed that higher values of serum biomarkers MMP-7, MMP-1, and periostin were found in patients with early fibrotic changes on CT scans [58]. These molecules are also described as potential biomarkers in IPF; thus, patients with this IPF-like phenotype may benefit from using antifibrotic drugs [74]. Several ongoing studies on ClinicalTrials.gov aim to assess the use of antifibrotics to prevent/attenuate the progression of pulmonary fibrosis after COVID-19 (NCT04607928, NCT04338802, NCT04541680, NCT04619680, NCT04856111, NCT04282902, NCT04653831) [75]. These trials could potentially lead to an individualized and tailored antifibrotic therapy.

Additionally, non-traditional treatment could help alleviate the symptoms of post-COVID-19 pulmonary fibrosis [76]. For example, treatment with epigallocatechin-3-gallate, the most abundant catechin in green tea, significantly inhibited irradiation-induced pulmonary fibrosis [77]. Other non-traditional options that have been studied for pulmonary fibrosis treatment, and that could be studied for post-COVID-19 pulmonary fibrosis treatment as well, are citrus extract [78], grape seed extract [79], rosemary extracts [80], plant glycosides [81], plant triterpene [82], polyphenols and flavonoids [83], alkaloids [84], and plant oils [85].

Although most of these options look promising, there is a clear obstacle to their use: the high cost of these therapeutic agents (ranging from USD 2500 to USD 7500 per month, dependent on the drug) [86,87]. In the best-case scenario, Ecuador is the Latin American country with the highest minimum monthly wage, USD 425 (Statista Research Group, 2022). It would be unrealistic for people to live on minimal monthly wage and pay for such treatment.

A study by Dempsey et al. in 2021 described that of all the patients with pulmonary fibrosis who were required to take Pirfenidone and Nintedanib, only 26.4% had started the medication, and half of them discontinued it. They concluded that adopting antifibrotic drugs in daily clinical practice is low and may be related to this therapy’s high costs [88]. Another similar study showed that the patients who used antifibrotics were mostly younger with greater severity of the disease, greater use of oxygen for physical activity, and more compromised quality of life [89].

For this reason, the challenge in addressing post-COVID-19 pulmonary fibrosis in Latin America goes beyond therapeutic alternatives. It involves the willingness of governments to facilitate access to effective medicines, and to create and improve access to multidisciplinary rehabilitation programs, which can care for all patients from Latin American countries that require intervention due to post-COVID-19 pulmonary consequences. Additionally, as mentioned previously, early detection may reduce the need for adherence to long-term treatment, a global issue exacerbated in Latin America by cultural and economic constraints [15,90].

Follow-up in centers with multidisciplinary teams and well-designed studies are necessary to develop scientific knowledge and a better understanding of long-COVID-19 and possible post-COVID-19 pulmonary fibrosis. It would help prevent disease worsening and long-term irreversible lung damage. We believe that raising awareness of the current challenges in Latin America when managing post-COVID-19 pulmonary fibrosis could aid in developing new guidelines and standardization, which may help us improve ’patients’ outcomes and avoid irreversible complications. The goal is to develop prevention, rehabilitation, and clinical management techniques that can be effectively implemented in Latin America. The following sections describe the barriers to the care of patients with post-COVID-19 pulmonary fibrosis in Latin America, and how these barriers can be addressed.

## 5. Low Resource Availability and Investment in Latin America

The first issue in addressing post-COVID-19 pulmonary fibrosis in Latin America is the inequity in the distribution of resources and health services, particularly in rural and underprivileged areas [91]. For example, it is estimated that 70% of 200 million people from underprivileged backgrounds do not have access to basic health care in the region [92]. Access to healthcare is further complicated by the public versus private healthcare systems common in Latin America [55]. Often, public healthcare systems are underfunded and overrun with patients needing care. At the same time, wealthier citizens can gain access to private institutions, creating greater inequity in care between people of different socioeconomic statuses.

Telemedicine appears to be a possible way to address these inequities in healthcare access. Due to the pandemic, teledigital approaches have rapidly replaced traditional in-person visits. Telemedicine has been widely adopted in the United States and Europe [93]. Physicians can easily conduct follow-ups with patients without requiring them to travel if they are infected with SARS-CoV-2 or have COVID-19 long-term sequelae. However, in Latin America, the adoption of telemedicine is lower in communities with high poverty levels [94,95].

Artificial intelligence (AI) has also already proven to be an excellent tool in the diagnosis and stratification of patients with pulmonary fibrosis (PF) [96]. Jiang et al. have proposed an AI-assisted chest high-resolution CT to evaluate the proportion of COVID-19 patients with PF [97]. Wang et al. also proposed using CT imaging and clinical data. Their AI system successfully predicted the time to critical illness for individual patients and identified high-risk patients. AI can potentially triage patients and accurately facilitate personalized treatment [98]. However, in Latin American countries, governments have not invested in these technologies.

## 6. Access to Multidisciplinary Teams

In addition to poor resource allocation and distribution as well as low investment in healthcare technologies across the region, Latin America also faces a scarcity of qualified healthcare professionals. One of the most significant challenges in follow-up with patients with post-COVID-19 lung lesions is the establishment of multidisciplinary teams (MDT) of healthcare professionals who carefully evaluate early symptoms or signs of disease progression and lack of improvement, while correctly identifying lung sequelae. Based on the consensus on pulmonary fibrosis, an MDT for evaluating and managing post-COVID-19 pulmonary fibrosis must include a pulmonologist, clinician, rehabilitator, cardiac consultant, physical therapist, occupational therapist, psychologist, neurologist, and other specialists according to the needs of the patients [41]. This MDT must be developed following standards and protocols supported by evidence for proper patient care and management.

The formation of these teams presents several challenges in Latin America because the number of physicians per population varies significantly between countries. Cuba has the highest number of physicians per capita, with 8.42 professionals per 1000 people in 2018, while Honduras has the fewest (0.31) per capita. Other Latin American countries have similarly low numbers of physicians, as evidenced by Costa Rica (2.89), Colombia (3.84), and Venezuela (1.73) [99].

Another barrier to forming these MDTs is the scarcity of medical specialists across the region. For example, in Mexico in 2018, it was estimated that there were only 750 Pulmonology specialists in the entire country [100]. Similarly, the Mexican Council of Neurology estimated that by 2021, there were only 1126 registered specialists in adult neurology [101], translating to approximately one neurologist per 100,000 inhabitants. Additionally, there are only 2.16 cardiologists per 100,000 inhabitants [100]. Similarly, Brazil had 1.53 pulmonologists per 100,000 people in 2014, and Panama had only 1.04 per 100,000 people in 2014.

The final and most alarming issue is that in Latin America, only half of the pulmonary rehabilitation (PR) centers evaluated dyspnea and fatigue because of the limited resources (as described above), despite being the most prevalent symptoms in post-COVID-19 patients. In 2021, less than 60% of the centers had a pulmonologist, and less than 40% had a cardiologist, psychiatrist, and clinicians on their teams. Mental health is also key to pulmonary rehabilitation. However, less than one-third of medical specialists (excluding psychiatrists) in Latin America do not feel prepared to assess depression in patients [95]. Furthermore, less than 50% of the institutions performed health-related quality of life (HRQoL) checks, spirometry assessments, and physical qualities and symptoms checks to assess lung function [102]. Another study by Cherrez et al. showed that while physicians had a positive attitude toward PR and considered it a relevant long-term care strategy following COVID-19, most conveyed that diagnosis and treatment of chronic pulmonary sequelae are unclear. Guidelines should be established for assessing pulmonary function [103].

According to the recommendations of the European Respiratory Society in coordination with the American Thoracic Society [104], patients with COVID-19 should have a formal assessment of physical and psychological functioning at 6–8 weeks post-discharge. This assessment includes physical check-ups (measure of respiratory function, exercise capacity), lung function using body plethysmography, electrocardiography, cardiac ultrasound, blood sampling, nutritional counseling, and questionnaires to assess psychological distress. These results encourage a comprehensive rehabilitation program, including endurance training, strength training, patient education, respiratory physiotherapy, daily living training, relaxation techniques, occupational therapy, psychological support, and nutritional counseling [104,105,106,107].

For the reasons described above, access to MDTs in Latin America for managing chronic pulmonary diseases, such as pulmonary fibrosis, is as low as 26.9% across the region [53]. MDTs are crucial to properly managing post-COVID-19 pulmonary fibrosis appropriately, and might also improve the quality of life of patients. However, the successful implementation of these MDTs in Latin American countries seems highly unlikely, considering patients’ limited access to pulmonologists and other specialists, and limited implementation of pulmonary rehabilitation practices.

## 7. Conclusions

This commentary aims to draw attention to the most significant challenges (along with some potential responses) that the Latin American region is currently facing in managing post-COVID-19 pulmonary fibrosis. Generally, there is a paucity of evidence supporting a successful long-term treatment for COVID-19 follow-up. This, coupled with inadequate medical equipment and a lack of resources, availability, and access to multidisciplinary treatment teams, contributes to a limited capacity to appropriately treat post-COVID-19 pulmonary fibrosis in Latin American countries. The commentary suggests that better distribution and investment of resources, improved infrastructure (primarily multicenter cohorts of COVID-19 survivors), and well-designed studies are required to develop scientific knowledge to improve treatment for the increasing prevalence of pulmonary fibrosis in Latin America. 

## Figures and Tables

**Table 1:** Potential therapeutic antifibrotics in post-COVID-19 pulmonary fibrosis.

Name	Mechanism of Action	Clinical Trial	Primary Outcomes
Pirfenidone	Impacts TGF-β and TGF-β-induced downstream mediators and products such as SMAD3; α-SMA; tenascin-c; fibronectin; collagen type I, II, and III; and the collagen-specific chaperone HSP47. Regulates growth factors, PDGF and bFGF, thereby modulating collagen production.Inhibits the expression of MMP-9, TIMP1, and MMP-2 directly or by reducing the synthesis of TGF-β and downstream mediators. Reduces TGF-β activation by MMPs [59]	FIBRO-COVID NCT04607928	Change from baseline % in FVC Change from baseline % fibrosis in HRCT of the lung
		PINCER NCT04856111	Change in the FVC (Time frame: 24 weeks)
Deupirfenidone	An orally bioavailable and deuterated form of Pirfenidone. Inhibits various proinflammatory mediators, such as IL-6, TNF-α, and TGF-β	NCT04652518	Change in 6MWT (Time frame: baseline to day 91)
Nintedanib	An ATP-competitive inhibitor of FGFR-1, VEGFR-2, and PDGFR-α and -β [60]	NCT04338802	Changes in FVC (Time frame: 8 weeks)
		ENDCOV-I NCT04619680	Change in FVC (Time frame: baseline and 180 days)
		PINCER NCT04856111	Change in FVC (Time frame: 24 weeks)
IN01 vaccine	Anti-EGF polyclonal neutralizing antibodies, thereby preventing the binding of this factor to its receptors, blocking the signaling of pathways related to the growth of tumor growth factor and fibrosis	COVINVAC NCT04537130	To evaluate the safety and tolerability of the IN01 vaccine in diagnosed ex-COVID-19 patients that develop fibrotic lung syndrome after infection
Colchicine	An anti-inflammatory drug that inhibits the synthesis of TNF-α, IL-6, monocyte migration, and secretion of MMP-9, all of these effects diminish the fibrotic impact of COVID-19 [61]	NCT04818489	Clinical status at week 2 Pulmonary fibrosis at week 2 Pulmonary fibrosis at 45 days
Treamid	A metal chelator that mediates inflammation reduction, and fibrosis reduces the deposition of hydroxyproline and collagen type I, fibronectin, and CTGF [62]	NCT04527354	Rate of clinically significant change in FVC and DLCO at week 4 relative to the baseline value Clinically significant changes include a relative ≥10% increase in FVC or a relative increase in FVC within the range from ≥5% to <10% and a relative ≥15% in DLCO
Fuzheng Huayu	FZHY drug serum can inhibit the expression of α–SMA and collagen synthesis in a dose-dependent manner [63]	NCT04279197	Improves the proportion of pulmonary fibrosis at week 24. Evaluation of pulmonary fibrosis; improvement judged by HRCT score. The improvement of lung function (FVC, FEV1, FVC/FEV1)

TGF-β = Transforming growth factor beta; HSP = heat shock protein; SMA  =  smooth muscle actin; PDGF = platelet-derived growth factor; bFGF = basic fibroblast growth factor; MMP-9 = matrix metalloproteinase 9; TIMP1 = tissue inhibitor of metalloproteinases 1; IL-6 = interleukin-6; TNF-α = tumor necrosis factor alpha; FGFR-1 = fibroblast growth factor receptor type 1; VEGFR-2 = vascular endothelial growth factor receptor type 2; EGF = epidermal growth factor; CTGF = connective tissue growth factor; FVC = forced vital capacity; HRCT = high-resolution computed tomography; 6MWT = six-minute walk test; DLCO = diffusing capacity of the lungs for carbon monoxide; FEV1 = forced expiratory volume in the first second; FVC/FEV1 = the Tiffeneau–Pinelli index, a calculated ratio used in the diagnosis of obstructive and restrictive lung disease.

## Data Availability

Not applicable.

## References

[1] Hui D.S., Zumla A. (2019). Severe Acute Respiratory Syndrome: Historical, Epidemiologic, and Clinical Features. Infect. Dis. Clin..

[2] Worobey M. (2021). Dissecting the Early COVID-19 Cases in Wuhan. Science.

[3] Abdelkafi I., Loukil S., Romdhane Y. (2022). Economic Uncertainty during COVID-19 Pandemic in Latin America and Asia. J. Knowl. Econ..

[4] Thakur B., Dubey P., Benitez J., Torres J.P., Reddy S., Shokar N., Aung K., Mukherjee D., Dwivedi A.K. (2021). A Systematic Review and Meta-Analysis of Geographic Differences in Comorbidities and Associated Severity and Mortality among Individuals with COVID-19. Sci. Rep..

[5] Statista Research Department Coronavirus En Latinoamérica: Países Con Más Casos|Statista. https://es.statista.com/estadisticas/1105121/numero-casos-covid-19-america-latina-caribe-pais/.

[6] Alkodaymi M.S., Omrani O.A., Fawzy N.A., Abou Shaar B., Almamlouk R., Riaz M., Obeidat M., Obeidat Y., Gerberi D., Taha R.M. (2022). Prevalence of Post-Acute COVID-19 Syndrome Symptoms at Different Follow-up Periods: A Systematic Review and Meta-Analysis. Clin. Microbiol. Infect..

[7] Cherrez-Ojeda I., Robles-Velasco K., Osorio M.F., Cottin V., Vergara Centeno J., Felix M. (2021). Follow-up of Two Cases of Suspected Interstitial Lung Disease Following Severe COVID-19 Infection Shows Persistent Changes in Imaging and Lung Function. Clin. Case Rep..

[8] Amin B.J.H., Kakamad F.H., Ahmed G.S., Ahmed S.F., Abdulla B.A., Mikael T.M., Salih R.Q., Salh A.M., Hussein D.A. (2022). Post COVID-19 Pulmonary Fibrosis; a Meta-Analysis Study. Ann. Med. Surg..

[9] Molina M. (2021). Phase-II Randomized Clinical Trial to Evaluate the Effect of Pirfenidone Compared to Placebo in Post-COVID19 Pulmonary Fibrosis. https://clinicaltrials.gov/ct2/show/NCT04607928.

[10] Dhooria S. (2022). A Study of the Efficacy and Safety of Pirfenidone vs. Nintedanib in the Treatment of Fibrotic Lung Disease After Coronavirus Disease-19 Pneumonia. https://clinicaltrials.gov/ct2/show/NCT04856111.

[11] PureTech (2021). A Phase 2 Randomized, Double-Blind, Placebo-Controlled Trial and Open Label Extension to Evaluate the Safety and Efficacy of Deupirfenidone (LYT-100) in Post-Acute COVID-19 Respiratory Disease. https://adisinsight.springer.com/trials/700330871.

[12] Zhang H. Efficacy and Safety of Nintedanib Ethanesulfonate Soft Capsule in the Treatment of Pulmonary Fibrosis in Patients With Moderate to Severe COVID-9(COVID 19): A Single-Center, Randomized, Placebo-Controlled Study.2020. https://adisinsight.springer.com/trials/700320667.

[13] Padilla M.L. (2022). Early Nintedanib Deployment in COVID-19 Interstitial Lung Disease. https://www.clinicalconnection.com/clinical-trials-from-other-databases/study-details-from-other-databases/551870/56298231/baylor-university-medical-center-dallas.

[14] Santos M.E., Villatoro P. (2018). A Multidimensional Poverty Index for Latin America. Rev. Income Wealth.

[15] Quijano-Ruiz A., Faytong-Haro M. (2021). Maternal Sexual Empowerment and Sexual and Reproductive Outcomes among Female Adolescents: Evidence from a Cross-Sectional Study in Ecuador. SSM—Popul. Health.

[16] Gasparini L., Lustig N. (2011). The Rise and Fall of Income Inequality in Latin America.

[17] Huber E., Nielsen F., Pribble J., Stephens J.D. (2006). Politics and Inequality in Latin America and the Caribbean. Am. Sociol. Rev..

[18] Eakin H.C., Wehbe M.B. (2009). Linking Local Vulnerability to System Sustainability in a Resilience Framework: Two Cases from Latin America. Clim. Chang..

[19] Liang L.-L., Tseng C.-H., Ho H.J., Wu C.-Y. (2020). Covid-19 Mortality Is Negatively Associated with Test Number and Government Effectiveness. Sci. Rep..

[20] Rojas D., Saavedra J., Petrova M., Pan Y., Szapocznik J. (2022). Predictors of COVID-19 Fatality: A Worldwide Analysis of the Pandemic over Time and in Latin America. J. Epidemiol. Glob. Health.

[21] Bahmer T., Borzikowsky C., Lieb W., Horn A., Krist L., Fricke J., Scheibenbogen C., Rabe K.F., Maetzler W., Maetzler C. (2022). Severity, Predictors and Clinical Correlates of Post-COVID-19 Syndrome (PCS) in Germany: A Prospective, Multi-Centre, Population-Based Cohort Study. EClinicalMedicine.

[22] CDC Post-COVID-19 Conditions. https://www.cdc.gov/coronavirus/2019-ncov/long-term-effects/index.html.

[23] Bakhoum M.F., Ritter M., Garg A.K., Chan A.X., Bakhoum C.Y., Smith D.M. (2022). Subclinical Ocular Inflammation in Persons Recovered from Ambulatory COVID-19 2020, 2020.09.22.20128140. medRxiv.

[24] Wong A.W., Fidler L., Marcoux V., Johannson K.A., Assayag D., Fisher J.H., Hambly N., Kolb M., Morisset J., Shapera S. (2020). Practical Considerations for the Diagnosis and Treatment of Fibrotic Interstitial Lung Disease During the Coronavirus Disease 2019 Pandemic. Chest.

[25] Venkataraman T., Coleman C.M., Frieman M.B. (2017). Overactive Epidermal Growth Factor Receptor Signaling Leads to Increased Fibrosis after Severe Acute Respiratory Syndrome Coronavirus Infection. J. Virol..

[26] Guarnera A., Santini E., Podda P. (2021). Idiopathic Interstitial Pneumonias and COVID-19 Pneumonia: Review of the Main Radiological Features and Differential Diagnosis. Tomography.

[27] Han X., Fan Y., Alwalid O., Zhang X., Jia X., Zheng Y., Shi H. (2021). Fibrotic Interstitial Lung Abnormalities at 1-Year Follow-up CT after Severe COVID-19. Radiology.

[28] Damiani S., Fiorentino M., De Palma A., Foschini M.P., Lazzarotto T., Gabrielli L., Viale P.L., Attard L., Riefolo M., D’Errico A. (2021). Pathological Post-mortem Findings in Lungs Infected with SARS-CoV-2. J. Pathol..

[29] Barnes B.J., Adrover J.M., Baxter-Stoltzfus A., Borczuk A., Cools-Lartigue J., Crawford J.M., Daßler-Plenker J., Guerci P., Huynh C., Knight J.S. (2020). Targeting Potential Drivers of COVID-19: Neutrophil Extracellular Traps. J. Exp. Med..

[30] Merza M.Y., Hwaiz R.A., Hamad B.K., Mohammad K.A., Hama H.A., Karim A.Y. (2021). Analysis of Cytokines in SARS-CoV-2 or COVID-19 Patients in Erbil City, Kurdistan Region of Iraq. PLoS ONE.

[31] Aesif S.W., Bribriesco A.C., Yadav R., Nugent S.L., Zubkus D., Tan C.D., Mehta A.C., Mukhopadhyay S. (2021). Pulmonary Pathology of COVID-19 Following 8 Weeks to 4 Months of Severe Disease: A Report of Three Cases, Including One with Bilateral Lung Transplantation. Am. J. Clin. Pathol..

[32] Bae I.-G., Hong K.-W., Yang J.W., Moon K., Kim J.D., Ju S., Cho M.-C. (2020). Persistent Pneumonic Consolidations Due to Secondary Organizing Pneumonia in a Patient Recovering from COVID-19 Pneumonia: A Case Report.

[33] Bazdyrev E., Rusina P., Panova M., Novikov F., Grishagin I., Nebolsin V. (2021). Lung Fibrosis after COVID-19: Treatment Prospects. Pharmaceuticals.

[34] Aul R., Gates J., Draper A., Dunleavy A., Ruickbie S., Meredith H., Walters N., van Zeller C., Taylor V., Bridgett M. (2021). Complications after Discharge with COVID-19 Infection and Risk Factors Associated with Development of Post-COVID-19 Pulmonary Fibrosis. Respir. Med..

[35] Ali R.M.M., Ghonimy M.B.I. (2021). Post-COVID-19 Pneumonia Lung Fibrosis: A Worrisome Sequelae in Surviving Patients. Egypt. J. Radiol. Nucl. Med..

[36] Ojo A.S., Balogun S.A., Williams O.T., Ojo O.S. (2020). Pulmonary Fibrosis in COVID-19 Survivors: Predictive Factors and Risk Reduction Strategies. Pulm. Med..

[37] Cocconcelli E., Bernardinello N., Giraudo C., Castelli G., Giorgino A., Leoni D., Petrarulo S., Ferrari A., Saetta M., Cattelan A. (2021). Characteristics and Prognostic Factors of Pulmonary Fibrosis after COVID-19 Pneumonia. Front. Med..

[38] Burki T. (2020). COVID-19 in Latin America. Lancet Infect. Dis..

[39] Aissaoui H., Eskenazi A., Suteau V., Adenis A., Drak Alsibai K. (2021). Case Report: Potential Role of Corticosteroids in the Management of Post-COVID-19 Pneumonia. Front. Med..

[40] Mohammadi A., Balan I., Yadav S., Matos W.F., Kharawala A., Gaddam M., Sarabia N., Koneru S.C., Suddapalli S.K., Marzban S. (2022). Post-COVID-19 Pulmonary Fibrosis. Cureus.

[41] Andrejak C., Cottin V., Crestani B., Debieuvre D., Gonzalez-Bermejo J., Morelot-Panzini C., Stach B., Uzunhan Y., Maitre B., Raherison C. (2020). Guide for Management of Patients with Possible Respiratory Sequelae after a SARS-CoV-2 Pneumonia. Support Proposals Developed by the French-Speaking Respiratory Medicine Society. Version of 10 November 2020. Rev. Mal. Respir..

[42] Ranu H., Wilde M., Madden B. (2011). Pulmonary Function Tests. Ulster Med. J..

[43] Yin X., Min X., Nan Y., Feng Z., Li B., Cai W., Xi X., Wang L. (2020). Assessment of the Severity of Coronavirus Disease: Quantitative Computed Tomography Parameters versus Semiquantitative Visual Score. Korean J. Radiol..

[44] Wu X., Liu X., Zhou Y., Yu H., Li R., Zhan Q., Ni F., Fang S., Lu Y., Ding X. (2021). 3-Month, 6-Month, 9-Month, and 12-Month Respiratory Outcomes in Patients Following COVID-19-Related Hospitalisation: A Prospective Study. Lancet Respir. Med..

[45] Carfì A., Bernabei R., Landi F. (2020). Against COVID-19. Post-Acute Care Study Group: For the Gemelli Against CCOVID-19 Post-Acute Care Study Group. Persistent Symptoms in Patients after Acute COVID-19. JAMA.

[46] Mandal S., Barnett J., Brill S.E., Brown J.S., Denneny E.K., Hare S.S., Heightman M., Hillman T.E., Jacob J., Jarvis H.C. (2021). ‘Long-COVID’: A Cross-Sectional Study of Persisting Symptoms, Biomarker and Imaging Abnormalities Following Hospitalisation for COVID-19. Thorax.

[47] Zha L., Shen Y., Pan L., Han M., Yang G., Teng X., Tefsen B. (2021). Follow-up Study on Pulmonary Function and Radiological Changes in Critically Ill Patients with COVID-19. J. Infect..

[48] Ngai J.C., Ko F.W., Ng S.S., TO K., Tong M., Hui D.S. (2010). The Long-term Impact of Severe Acute Respiratory Syndrome on Pulmonary Function, Exercise Capacity and Health Status. Respirology.

[49] Sanyaolu A., Marinkovic A., Prakash S., Zhao A., Balendra V., Haider N., Jain I., Simic T., Okorie C. (2022). Post-Acute Sequelae in COVID-19 Survivors: An Overview. SN Compr. Clin. Med..

[50] Vázquez-García J.C., Pérez-Padilla R., Casas A., Schönffeldt-Guerrero P., Pereira J., Vargas-Domínguez C., Velázquez-Uncal M., Martínez-Briseño D., Torre-Bouscoulet L., Gochicoa-Rangel L. (2016). Reference Values for the Diffusing Capacity Determined by the Single-Breath Technique at Different Altitudes: The Latin American Single-Breath Diffusing Capacity Reference Project. Respir. Care.

[51] Michalski J.E., Kurche J.S., Schwartz D.A. (2021). From ARDS to Pulmonary Fibrosis: The next Phase of the COVID-19 Pandemic?. Transl. Res..

[52] Franquet T., Giménez A., Ketai L., Mazzini S., Rial A., Pomar V., Domingo P. (2022). Air Trapping in COVID-19 Patients Following Hospital Discharge: Retrospective Evaluation with Paired Inspiratory/Expiratory Thin-Section CT. Eur. Radiol..

[53] Cherrez-Ojeda I., Cottin V., Calderón J.C., Delgado C., Calero E., Simanca-Racines D., Quadrelli S., Cherrez A. (2018). Management and Attitudes about IPF (Idiopathic Pulmonary Fibrosis) among Physicians from Latin America. BMC Pulm. Med..

[54] Lopes A.J., Litrento P.F., Provenzano B.C., Carneiro A.S., Monnerat L.B., da Cal M.S., Ghetti A.T.A., Mafort T.T. (2021). Small Airway Dysfunction on Impulse Oscillometry and Pathological Signs on Lung Ultrasound Are Frequent in Post-COVID-19 Patients with Persistent Respiratory Symptoms. PLoS ONE.

[55] Homedes N., Ugalde A. (2005). Why Neoliberal Health Reforms Have Failed in Latin America. Health Policy.

[56] Lechowicz K., Drożdżal S., Machaj F., Rosik J., Szostak B., Zegan-Barańska M., Biernawska J., Dabrowski W., Rotter I., Kotfis K. (2020). COVID-19: The Potential Treatment of Pulmonary Fibrosis Associated with SARS-CoV-2 Infection. J. Clin. Med..

[57] Myall K.J., Mukherjee B., Castanheira A.M., Lam J.L., Benedetti G., Mak S.M., Preston R., Thillai M., Dewar A., Molyneaux P.L. (2021). Persistent Post–COVID-19 Interstitial Lung Disease. An Observational Study of Corticosteroid Treatment. Ann. Am. Thorac. Soc..

[58] Safont B., Tarraso J., Rodriguez-Borja E., Fernández-Fabrellas E., Sancho-Chust J.N., Molina V., Lopez-Ramirez C., Lope-Martinez A., Cabanes L., Andreu A.L. (2022). Lung Function, Radiological Findings and Biomarkers of Fibrogenesis in a Cohort of COVID-19 Patients Six Months After Hospital Discharge. Arch. Bronconeumol..

[59] Ruwanpura S.M., Thomas B.J., Bardin P.G. (2020). Pirfenidone: Molecular Mechanisms and Potential Clinical Applications in Lung Disease. Am. J. Respir. Cell Mol. Biol..

[60] Wollin L., Wex E., Pautsch A., Schnapp G., Hostettler K.E., Stowasser S., Kolb M. (2015). Mode of Action of Nintedanib in the Treatment of Idiopathic Pulmonary Fibrosis. Eur. Respir. J..

[61] Issak E.R. (2021). Impact of Colchicine on the Clinical Outcome of COVID-19 and the Development of Post-COVID-19 Pulmonary Fibrosis: Randomized Controlled Clinical Trial. clinicaltrials.gov.

[62] Skurikhin E., Nebolsin V., Widera D., Ermakova N., Pershina O., Pakhomova A., Krupin V., Pan E., Zhukova M., Novikov F. (2020). Antifibrotic and Regenerative Effects of Treamid in Pulmonary Fibrosis. Int. J. Mol. Sci..

[63] Chenghai L. (2021). Efficacy and Safety of Fuzheng Huayu Tablets in Post-COVID-19 Patients With Pulmonary Inflammation and Fibrosis: A Multicenter Double-Blind Randomized Controlled Trial. https://ichgcp.net/clinical-trials-registry/NCT04279197.

[64] Vitiello A., Pelliccia C., Ferrara F. (2020). COVID-19 Patients with Pulmonary Fibrotic Tissue: Clinical Pharmacological Rational of Antifibrotic Therapy. SN Compr. Clin. Med..

[65] King Jr T.E., Bradford W.Z., Castro-Bernardini S., Fagan E.A., Glaspole I., Glassberg M.K., Gorina E., Hopkins P.M., Kardatzke D., Lancaster L. (2014). A Phase 3 Trial of Pirfenidone in Patients with Idiopathic Pulmonary Fibrosis. N. Engl. J. Med..

[66] Richeldi L., Du Bois R.M., Raghu G., Azuma A., Brown K.K., Costabel U., Cottin V., Flaherty K.R., Hansell D.M., Inoue Y. (2014). Efficacy and Safety of Nintedanib in Idiopathic Pulmonary Fibrosis. N. Engl. J. Med..

[67] Kotfis K. (2021). The Use of a Mineralocorticoid Receptor Antagonist (Spironolactone) in the Treatment of Pulmonary Fibrosis Associated With SARS-CoV-2 Infection. https://clinicaltrials.gov/ct2/show/NCT04912011.

[68] NPO Petrovax (2021). Multicenter, Open-Label Prospective Cohort Study of the Efficacy and Safety of the Inclusion of Longidaze in the Prevention and Treatment of Post-Inflammatory Pulmonary Fibrosis and Interstitial Lung Diseases Caused by COVID-19. https://www.researcher-app.com/paper/6408795.

[69] Guy’s and St Thomas’ NHS Foundation Trust (2021). Phase I/II MONACO Cell Therapy Study: Monocytes as an Antifibrotic Treatment After COVID-19. https://www.medifind.com/articles/clinical-trial/255656033.

[70] Humanetics Corporation (2022). A Phase 2 Study of BIO 300 Oral Suspension in Discharged COVID-19 Patients. https://www.niaid.nih.gov/clinical-trials/phase-2-study-bio-300-oral-suspension-discharged-covid-19-patients.

[71] Rashad A. (2020). Short Term Low Dose Corticosteroids for Management of Post Covid-19 Pulmonary Fibrosis. https://clinicaltrials.gov/ct2/show/NCT04551781.

[72] Vitti Labs, LLC (2022). Safety and Effectiveness of EV-Pure + WJ-Pure Treatment on Pulmonary Fibrosis Secondary to Covid-19. https://clinicaltrials.gov/ct2/show/NCT05387239.

[73] Instituto Oncológico Dr Rosell (2020). Phase Ib Controlled Exploratory Trial for Treatment of Fibrosing Interstitial Lung Disease Patients Secondary to SARS-CoV-2 Infection With IN01 Vaccine (COVINVAC). https://data.cochrane.org/concepts/GEn3ooKangHqBO.

[74] Rosas I.O., Richards T.J., Konishi K., Zhang Y., Gibson K., Lokshin A.E., Lindell K.O., Cisneros J., MacDonald S.D., Pardo A. (2008). MMP1 and MMP7 as Potential Peripheral Blood Biomarkers in Idiopathic Pulmonary Fibrosis. PLoS Med..

[75] Clinical Trials Home—ClinicalTrials.Gov. https://clinicaltrials.gov/.

[76] Bahri S., Ali R.B., Abidi A., Jameleddine S. (2017). The Efficacy of Plant Extract and Bioactive Compounds Approaches in the Treatment of Pulmonary Fibrosis: A Systematic Review. Biomed. Pharmacother..

[77] You H., Wei L., Sun W.-L., Wang L., Yang Z.-L., Liu Y., Zheng K., Wang Y., Zhang W.-J. (2014). The Green Tea Extract Epigallocatechin-3-Gallate Inhibits Irradiation-Induced Pulmonary Fibrosis in Adult Rats. Int. J. Mol. Med..

[78] Zhou X.-M., Huang M.-M., He C.-C., Li J.-X. (2009). Inhibitory Effects of Citrus Extracts on the Experimental Pulmonary Fibrosis. J. Ethnopharmacol..

[79] Nuttall S., Kendall M., Bombardelli E., Morazzoni P. (1998). An Evaluation of the Antioxidant Activity of a Standardized Grape Seed Extract, Leucoselect^®^. J. Clin. Pharm. Ther..

[80] Yang L.-T., Liu X., Cheng D.-Y., Fang X., Mu M., Hu X.-B., Nie L. (2013). Effects of Diterpene Phenol Extract of Rosmarinus Officinalis on TGFbeta1 and MRNA Expressions of Its Signaling Pathway Molecules in the Lung Tissue of Pulmonary Fibrosis Rats. Zhongguo Zhong Xi Yi Jie He Za Zhi Zhongguo Zhongxiyi Jiehe Zazhi Chin. J. Integr. Tradit. West. Med..

[81] Kandhare A.D., Bodhankar S.L., Mohan V., Thakurdesai P.A. (2015). Effect of Glycosides Based Standardized Fenugreek Seed Extract in Bleomycin-Induced Pulmonary Fibrosis in Rats: Decisive Role of Bax, Nrf2, NF-ΚB, Muc5ac, TNF-α and IL-1β. Chem. Biol. Interact..

[82] Yang Y., Huang Y., Huang C., Lv X., Liu L., Wang Y., Li J. (2012). Antifibrosis Effects of Triterpene Acids of Eriobotrya Japonica (Thunb.) Lindl. Leaf in a Rat Model of Bleomycin-Induced Pulmonary Fibrosis. J. Pharm. Pharmacol..

[83] Impellizzeri D., Talero E., Siracusa R., Alcaide A., Cordaro M., Zubelia J.M., Bruschetta G., Crupi R., Esposito E., Cuzzocrea S. (2015). Protective Effect of Polyphenols in an Inflammatory Process Associated with Experimental Pulmonary Fibrosis in Mice. Br. J. Nutr..

[84] Ma X., Chen R., Liu X., Xie J., Si K., Duan L. (2013). Effects of Matrine on JAK-STAT Signaling Transduction Pathways in Bleomycin-Induced Pulmonary Fibrosis. Afr. J. Tradit. Complement. Altern. Med..

[85] Abidi A., Serairi R., Kourda N., Ben Ali R., Ben Khamsa S., Feki M. (2016). Therapeutic Effect of Flaxseed Oil on Experimental Pulmonary Fibrosis Induced by Bleomycin in Rats. Eur. J. Inflamm..

[86] Pirfenidona (Esbriet) y Nintedanib (Ofev) Para La Fibrosis Pulmonar Idiopática—Info-Farmacia. http://www.info-farmacia.com/medico-farmaceuticos/informes-tecnicos/pirfenidona-esbriet-y-nintedanib-ofev-para-la-fibrosis-pulmonar-idiopatica.

[87] Feldman D.J. (2015). OFEV (Nintedanib) to Treat Idiopathic Pulmonary Fibrosis. Idiopathic Pulm. Fibros..

[88] Dempsey T.M., Payne S., Sangaralingham L., Yao X., Shah N.D., Limper A.H. (2021). Adoption of the Antifibrotic Medications Pirfenidone and Nintedanib for Patients with Idiopathic Pulmonary Fibrosis. Ann. Am. Thorac. Soc..

[89] Salisbury M.L., Conoscenti C.S., Culver D.A., Yow E., Neely M.L., Bender S., Hartmann N., Palmer S.M., Leonard T.B. (2020). Antifibrotic Drug Use in Patients with Idiopathic Pulmonary Fibrosis. Data from the IPF-PRO Registry. Ann. Am. Thorac. Soc..

[90] Holland M., Vieira F.V., Canuto O., Holland M., Vieira F.V., Canuto O. (2004). Economic Growth and the Balance-of-Payments Constraint in Latin America. Investig. Económica.

[91] Wallace S.P., Gutiérrez V.F. (2005). Equity of Access to Health Care for Older Adults in Four Major Latin American Cities. Rev. Panam. Salud Pública.

[92] International Labour Organization More than 140 Million Denied Access to Health Care in Latin America and the Caribbean. http://www.ilo.org/global/about-the-ilo/newsroom/news/WCMS_007961/lang--en/index.htm.

[93] Ye S., Kronish I., Fleck E., Fleischut P., Homma S., Masini D., Moise N. (2021). Telemedicine Expansion During the COVID-19 Pandemic and the Potential for Technology-Driven Disparities. J. Gen. Intern. Med..

[94] Cantor J.H., McBain R.K., Pera M.F., Bravata D.M., Whaley C.M. (2021). Who Is (and Is Not) Receiving Telemedicine Care During the COVID-19 Pandemic. Am. J. Prev. Med..

[95] Camacho-Leon G., Faytong-Haro M., Carrera K., Molero M., Melean F., Reyes Y., Mautong H., De La Hoz I., Cherrez-Ojeda I. (2022). A Narrative Review of Telemedicine in Latin America during the COVID-19 Pandemic.

[96] Christe A., Peters A.A., Drakopoulos D., Heverhagen J.T., Geiser T., Stathopoulou T., Christodoulidis S., Anthimopoulos M., Mougiakakou S.G., Ebner L. (2019). Computer-Aided Diagnosis of Pulmonary Fibrosis Using Deep Learning and CT Images. Investig. Radiol..

[97] Zou J.-N., Sun L., Wang B.-R., Zou Y., Xu S., Ding Y.-J., Shen L.-J., Huang W.-C., Jiang X.-J., Chen S.-M. (2021). The Characteristics and Evolution of Pulmonary Fibrosis in COVID-19 Patients as Assessed by AI-Assisted Chest HRCT. PLoS ONE.

[98] Wang R., Jiao Z., Yang L., Choi J.W., Xiong Z., Halsey K., Tran T.M.L., Pan I., Collins S.A., Feng X. (2022). Artificial Intelligence for Prediction of COVID-19 Progression Using CT Imaging and Clinical Data. Eur. Radiol..

[99] The World Bank Physicians (per 1000 People)|Data. https://data.worldbank.org/indicator/SH.MED.PHYS.ZS.

[100] Torre-Bouscoulet L. (2018). Especialistas líderes en Medicina Respiratoria. Neumol. Cir. Tórax.

[101] Consejo Mexicano de Neurología Neurólogos Certificados|Consejo Mexicano de Neurología. https://www.consejomexicanodeneurologia.org/neurologos-certificados/.

[102] Benavides-Cordoba V., Barros-Poblete M., Vieira R.P., Mazzucco G., Fregonezi G., Torres-Castro R. (2022). Provision of Pulmonary Rehabilitation in Latin America 18 Months after the COVID-19 Pandemic: A Survey of the Latin American Thoracic Association. Chron. Respir. Dis..

[103] Cherrez-Ojeda I., Vanegas E., Felix M., Farfán Bajaña M.J., Sarfraz A., Sarfraz Z., Camacho G., Barrios-Ruiz A., Michel J. (2022). Physician’s Attitudes on Pulmonary Rehabilitation Following COVID-19: A Brief Perspective from a Developing Country. Multidiscip. Respir. Med..

[104] Spruit M.A., Holland A.E., Singh S.J., Tonia T., Wilson K.C., Troosters T. (2020). COVID-19: Interim Guidance on Rehabilitation in the Hospital and Post-Hospital Phase from a European Respiratory Society- and American Thoracic Society-Coordinated International Task Force. Eur. Respir. J..

[105] Gloeckl R., Leitl D., Jarosch I., Schneeberger T., Nell C., Stenzel N., Vogelmeier C.F., Kenn K., Koczulla A.R. (2021). Benefits of Pulmonary Rehabilitation in COVID-19: A Prospective Observational Cohort Study. ERJ Open Res..

[106] Barker-Davies R.M., O’Sullivan O., Senaratne K.P.P., Baker P., Cranley M., Dharm-Datta S., Ellis H., Goodall D., Gough M., Lewis S. (2020). The Stanford Hall Consensus Statement for Post-COVID-19 Rehabilitation. Br. J. Sports Med..

[107] Aytür Y.K., Köseoğlu B.F., Taşkıran Ö.Ö., Ordu-Gökkaya N.K., Delialioğlu S.Ü., Tur B.S., Sarıkaya S., Şirzai H., Tiftik T.T., Alemdaroğlu E. (2020). Pulmonary Rehabilitation Principles in SARS-COV-2 Infection (COVID-19): A Guideline for the Acute and Subacute Rehabilitation. Turk. J. Phys. Med. Rehabil..

